# Quantum key distribution with chromatic codes

**DOI:** 10.1038/s41377-025-01781-6

**Published:** 2025-03-19

**Authors:** Zhihui Yan, Xiaojun Jia

**Affiliations:** State Key Laboratory of Quantum Optics and Quantum Optics Devices, Institute of Opto-Electronics, Collaborative Innovation Center of Extreme Optics, Shanxi University, Taiyuan, China

**Keywords:** Quantum optics, Quantum optics

## Abstract

Quantum key distribution with different frequency codes is demonstrated with a reconfigurable entanglement distribution network, which is essential for scalable and resource-efficient quantum communications.

Quantum key distribution (QKD) enables communication security to outperform the classical approach^1^. Compared with prepare-and-measure schemes, quantum entanglement is a core resource of quantum information science and can enhance security against coherent attacks^2^. With the development of quantum technology, there is an increasing demand for the construction of large-scale quantum networks involving a growing number of users over long distance for the secure communication^3,4^. It is challenging to develop the scalability of entanglement-based QKD, due to the issues arising from distance limitations, degraded security in the face of advanced attacks, resource overhead, and stabilization techniques. In particular, the frequency degree of freedom is less susceptible to decoherence, and thus frequency-encoded signals can be transmitted in parallel without significant loss of quality and coherence^5–7^. To date, the hybrid encoding scheme with frequency degrees of freedom and time-bin encoding has been used in QKD protocols^8–10^. However, bulky interferometric setups with phase stabilization are needed, which suffer from a lack of reconfigurability and scalability issues.

For practical quantum communication networks, in a newly published paper in Light: Science & Applications, Anahita Khodadad Kashi and Michael Kues from the Institute of Photonics at Leibniz University Hannover reported an experimental demonstration of frequency-bin-encoded QKD in a reconfigurable entanglement distribution network^11^. The technique of a scalable frequency-bin basis analyzer module is developed, which allows for passive random basis selection, and a single detector for each user rather than four detectors, which reduces system complexity and hardware overhead, as shown in Fig. 1. The frequency-bin encoded BBM92 QKD protocol with a scalable frequency-bin basis analyzer module is demonstrated. Furthermore, this system enables a reconfigurable multiuser quantum communication network with adaptive frequency multiplexing capability without hardware overhead. These results reduce system complexity while maintaining security and performance to support a growing number of users. The quantum frequency complex technique plays an essential role in large-scale quantum communication networks.

**Fig. 1:**
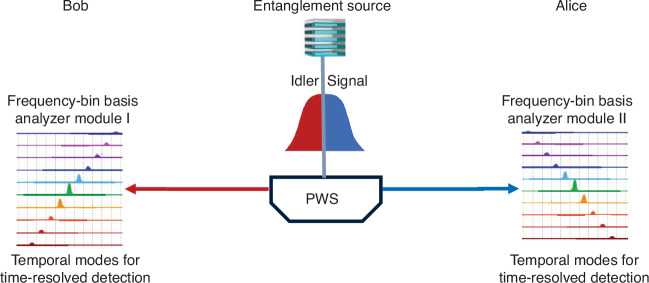
Schematic view of the frequency-bin-encoded entanglement-based quantum key distribution. Alice and Bob users are connected with signal and idler entangled spectra, which are generated from an entanglement source with a programmable wavelength switch (PWS). The frequency-bin basis analyzer module is developed for processing the spectra. First, the projection measurements are displayed in the frequency mixer stage. Then, the frequency-to-time mapping unit projects the phase-modulated spectral bins to the distinct temporal modes, corresponding to frequency bins under negative dispersion. Finally, time-resolved detection of the frequency-mixed spectrum is implemented

The quantum network holds the potential to inspire advancements in both quantum technology and QKD applications, as illustrated in Fig. 2. A high rate, long distance and high security are needed in QKD, besides the large number of channels, as discussed in this work. The rate of photonics QKD can be increased by increasing the repetition rate, the fidelity of quantum state encoding and decoding, and detector performance^12–14^. In addition to the above discrete variable high-rate QKD, the continuous variable quantum information system enables high-rate quantum communications in the metropolitan area, with the advantages of high-efficiency generation and detection^15–18^. Experimental demonstrations of long-distance QKD have been achieved through free space and fiber channels^19–21^. Device-independent quantum key distribution can distribute secret keys using untrusted devices, which offering not only information-theoretic security against channel attacks, but also against attacks exploiting implementation loopholes^22^. Furthermore, measurement-device-independent QKD, as a simple approach, can remove the most critical part of the implementation in all detections with both excellent security and performance^23^. Security can be guaranteed against individual attacks in a semi-device-independent scenario^24,25^. In the future, the hybrid architecture of these technologies provides a possible way for a high-performance QKD network. While the quantum network inspires future possible applications, such as quantum teleportation^26^, it also advances related quantum technologies.

**Fig. 2:**
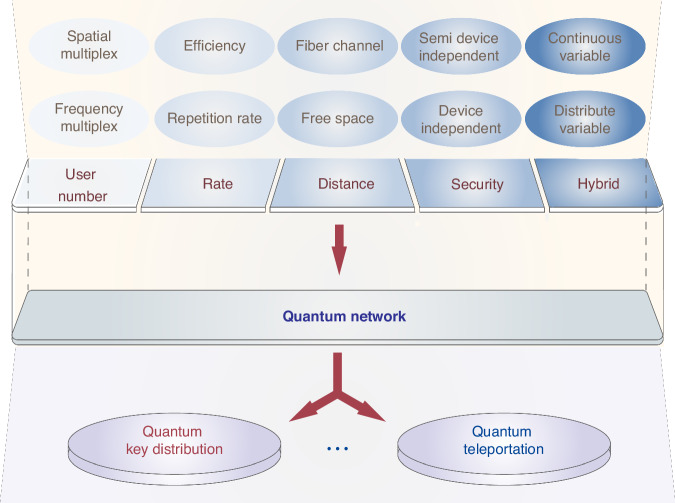
Future vision of the quantum network for quantum key distribution
